# Control of Variant Surface Glycoprotein Expression by CFB2 in Trypanosoma brucei and Quantitative Proteomic Connections to Translation and Cytokinesis

**DOI:** 10.1128/msphere.00069-22

**Published:** 2022-03-21

**Authors:** Gustavo Bravo Ruiz, Michele Tinti, Melanie Ridgway, David Horn

**Affiliations:** a The Wellcome Trust Centre for Anti-Infectives Research, School of Life Sciences, University of Dundee, Dundee, United Kingdom; University at Buffalo

**Keywords:** *Trypanosoma brucei*, mRNA binding, proteomics, RNAi

## Abstract

Variant surface glycoproteins (VSGs) coat parasitic African trypanosomes and underpin antigenic variation and immune evasion. These VSGs are superabundant virulence factors that are subject to posttranscriptional gene expression controls mediated via the *VSG* 3′ untranslated region (UTR). To identify positive VSG regulators in bloodstream-form Trypanosoma brucei, we used genome-scale screening data to prioritize mRNA binding protein (mRBP) knockdowns that phenocopy VSG mRNA knockdown, displaying loss of fitness and precytokinesis accumulation. The top three candidates were CFB2 (cyclin F-box protein 2) (Tb927.1.4650), MKT1 (Tb927.6.4770), and PBP1 (polyadenylate binding protein 1) (Tb927.8.4540). Notably, CFB2 was recently found to regulate VSG transcript stability, and all three proteins were found to associate. We used data-independent acquisition for accurate label-free quantification and deep proteome coverage to quantify the expression profiles following the depletion of each mRBP. Only CFB2 knockdown significantly reduced VSG expression and the expression of a reporter under the control of the *VSG* 3′ UTR. CFB2 knockdown also triggered the depletion of cytoplasmic ribosomal proteins, consistent with translation arrest observed when VSG synthesis is blocked. In contrast, PBP1 knockdown triggered the depletion of CFB2, MKT1, and other components of the PBP1 complex. Finally, all three knockdowns triggered the depletion of cytokinesis initiation factors, consistent with a cytokinesis defect, which was confirmed here for all three knockdowns. Thus, genome-scale knockdown data sets facilitate the triage and prioritization of candidate regulators. Quantitative proteomic analysis confirms the 3′-UTR-dependent positive control of VSG expression by CFB2 and interactions with additional mRBPs. Our results also reveal new insights into the connections between VSG expression control by CFB2, ribosomal protein expression, and cytokinesis.

**IMPORTANCE** VSG expression represents a key parasite virulence mechanism and an example of extreme biology. Posttranscriptional gene expression controls in trypanosomatids also continue to be the subject of substantial research interest. We have identified three candidate VSG regulators and used knockdown and quantitative proteomics, in combination with other approaches, to assess their function. CFB2 is found to control VSG expression via the *VSG* 3′ untranslated region, while other data support the view that MKT1 and PBP1 also form part of a CFB2 mRNA binding complex. Remarkably, we also find the depletion of cytoplasmic ribosomal proteins upon CFB2 knockdown, consistent with translation arrest observed when VSG synthesis is blocked. Proteomic profiles following knockdown further yield insights into cytokinesis defects. Taken together, our findings confirm and elaborate the role of CFB2 in controlling VSG expression and reveal new insights into connectivity with translation and cytokinesis controls.

## INTRODUCTION

African trypanosomes are parasitic protists that undergo antigenic variation to persist in immunocompetent mammalian hosts. Trypanosoma brucei is transmitted by tsetse flies and causes lethal and debilitating diseases in both humans and livestock. Antigenic variation involves the expression of a dense coat of superabundant variant surface glycoprotein (VSG) derived from a single VSG gene at a time (1). VSG expression can switch periodically to produce new VSG coats with a distinct set of epitopes. Thus, VSG underpins a key virulence mechanism, is encoded by the most abundant mRNA in the cell, and is the most abundant protein expressed by the cell. This extreme biology has been the source of a number of discoveries regarding gene expression controls, some of which have subsequently been found to be pervasive and to extend to other trypanosomatids. First, mRNAs encoding VSGs, and other mRNAs, were found to be *trans*-spliced (2), which was subsequently found to be the case for every trypanosomatid mRNA. Second, *VSG* genes were found to be part of a polycistronic transcription unit (3), which was subsequently found to be the case for almost all trypanosomatid genes. Third, *VSG* genes were found to be transcribed by RNA polymerase I (4), which was also found to be the case for other developmentally regulated genes.

Although *VSG* genes are subject to developmentally regulated transcription and *trans*-splicing control (5), other long polycistrons in trypanosomatids are constitutively transcribed and *trans*-spliced. As a consequence, posttranscriptional gene expression controls are pervasive, and gene expression is typically thought to be controlled by mRNA binding proteins (mRBPs) that, in most cases, bind the mRNA 3′ untranslated regions (UTRs). The VSGs are among only a few genes with a known regulatory motif within the mRNA 3′ UTR. In this case, a “16-mer” is required for high-level expression in the bloodstream form (6, 7).

Since VSG knockdown (8) or blocking VSG translation (9) results in a rapid growth defect and also specific precytokinesis cell cycle arrest, we reasoned that candidate VSG-positive regulators would be mRBPs associated with similar phenotypes in high-throughput knockdown screens (10, 11). We identified three candidates using this approach, CFB2 (cyclin F-box protein 2), MKT1, and PBP1 (polyadenylate binding protein 1), all of which were recently and independently found to stabilize and/or bind VSG mRNA (6). Using mRBP knockdown and quantitative proteomics, we confirm a specific role for CFB2 in VSG expression control. We also identify connections to other mRBP complex components, translation controls, and cytokinesis defects.

## RESULTS AND DISCUSSION

### Identification and assessment of candidate VSG regulators.

**FIG 1:**
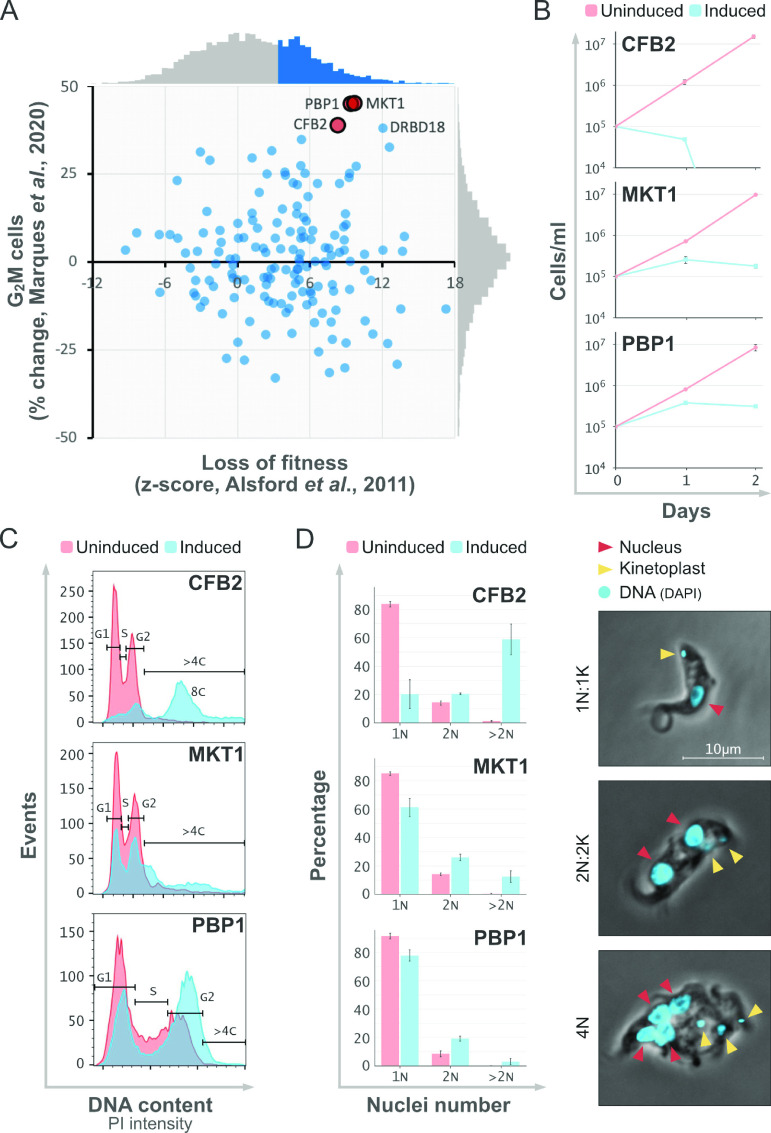
Identification and assessment of candidate VSG regulators. (A) Plot showing data for genes linked to the Gene Ontology term mRNA binding (GO:0003729) (*n* = 178) and in relation to loss of fitness and accumulation in G_2_M phase from RNA interference knockdown screens and from the sources indicated (10, 11). Genes with high scores in both screens are highlighted. The distribution of each full data set (>7,000 genes in each case) is shown beside each axis, where blue indicates a significant loss of fitness. (B) Growth curves for the knockdown strains showing cells per milliliter versus time. A total of 1 × 10^5^ cells/mL were grown in HMI-11 medium without TET (uninduced) or with TET (induced) to induce knockdown. Error bars represent standard errors (SE) from two independent experiments. (C) Cell cycle profiles for the knockdown strains as assessed by flow cytometry and for cells grown for 24 h without TET (uninduced) or with TET (induced). DNA was stained with propidium iodide (PI). Cell cycle phases (G_1_, S, and G_2_) and increased ploidy (>4C and 8C) are indicated. (D) Percentages of 1N (1 nucleus), 2N (postmitotic), and multinucleated (>2N) cells in the knockdown strains. Cells were grown as described above for panel C but with DAPI staining for DNA visualization. At least 100 cells were counted by microscopy using the DAPI and phase channels. Error bars represent SE for counts made by two independent observers. The images show examples of cells with 1 nucleus and 1 kinetoplast (1N:1K), 2 nuclei and 2 kinetoplasts (2N:2K), and 4 nuclei (4N). Arrows indicate DNA in nuclei or kinetoplasts.

The depletion of VSG mRNA in bloodstream-form T. brucei rapidly triggers precytokinesis cell cycle arrest (8). We reasoned that the depletion of mRNA binding proteins that positively control either VSG mRNA stability or translation would trigger a similar phenotype. To prioritize such candidate VSG regulators, we used genome-scale RNA interference (RNAi) screening data sets from bloodstream-form T. brucei, reporting a relative loss of fitness (10) or relative G_2_M accumulation precytokinesis (11). All T. brucei genes linked to the Gene Ontology term “mRNA binding” (GO:0003729) (*n* = 178) were assessed for these phenotypes, and the top three candidates were selected for further analysis (Fig. 1A). These three proteins were associated with a significant loss of fitness, with an average z-score of >8 (10) and with >35% increased G_2_M-phase accumulation (11). CFB2 is a cyclin-like F-box protein (Tb927.1.4650) previously shown to be required for cytokinesis in bloodstream-form T. brucei (12). MKT1 (Tb927.6.4770) is recruited to mRNAs by sequence-specific RNA binding proteins and stabilizes the bound mRNA, while PBP1 (Tb927.8.4540) interacts with MKT1 as well as with poly(A) binding protein 2 (13, 14); both MKT1 and PBP1 were recently found to bind VSG mRNA through CFB2 (6). DRBD18 also registered >35% increased G_2_M-phase accumulation, but this protein is known to promote nuclear mRNA export (15), so it was not investigated further here.

### Candidate RBP knockdowns trigger severe growth and cell cycle defects.

We constructed tetracycline (TET)-inducible RNAi strains, targeting either CFB2, MKT1, or PBP1 for knockdown. Each strain was grown in tetracycline, and growth was monitored for 48 h. Consistent with the loss of fitness reported previously in the genome-scale knockdown screen (10), we observed a severe growth defect in each case following knockdown (Fig. 1B). Loss of fitness was apparent by only 24 h, while CFB2 knockdown had the greatest impact. We next assessed each strain following 24 h of knockdown by flow cytometry and microscopy. Consistent with the overrepresentation of G_2_M-phase cells reported previously in the genome-scale knockdown screen (11), we observed an overrepresentation of 4C (G_2_-phase) cells following knockdown and as assessed by flow cytometry; ratios of 4C to 2C (G_1_-phase) plus S-phase cells were increased 3-, 1.3-, and 3.3-fold following CFB2, MKT1, and PBP1 knockdown, respectively (Fig. 1C). Each knockdown generated a distinct profile, however, with MKT1 knockdown increasing the DNA content above 4C and CFB2 knockdown, in particular, also yielding cells that endoreduplicated their DNA in the absence of cytokinesis, producing a high proportion of 8C cells (Fig. 1C). Indeed, microscopic examination revealed approximately 60% abnormal multinucleated cells following CFB2 knockdown, indicating that mitosis continued in these cells in the absence of cytokinesis (Fig. 1D).

### Quantitative proteomic profiles reveal distinct responses to RBP knockdown.

**FIG 2:**
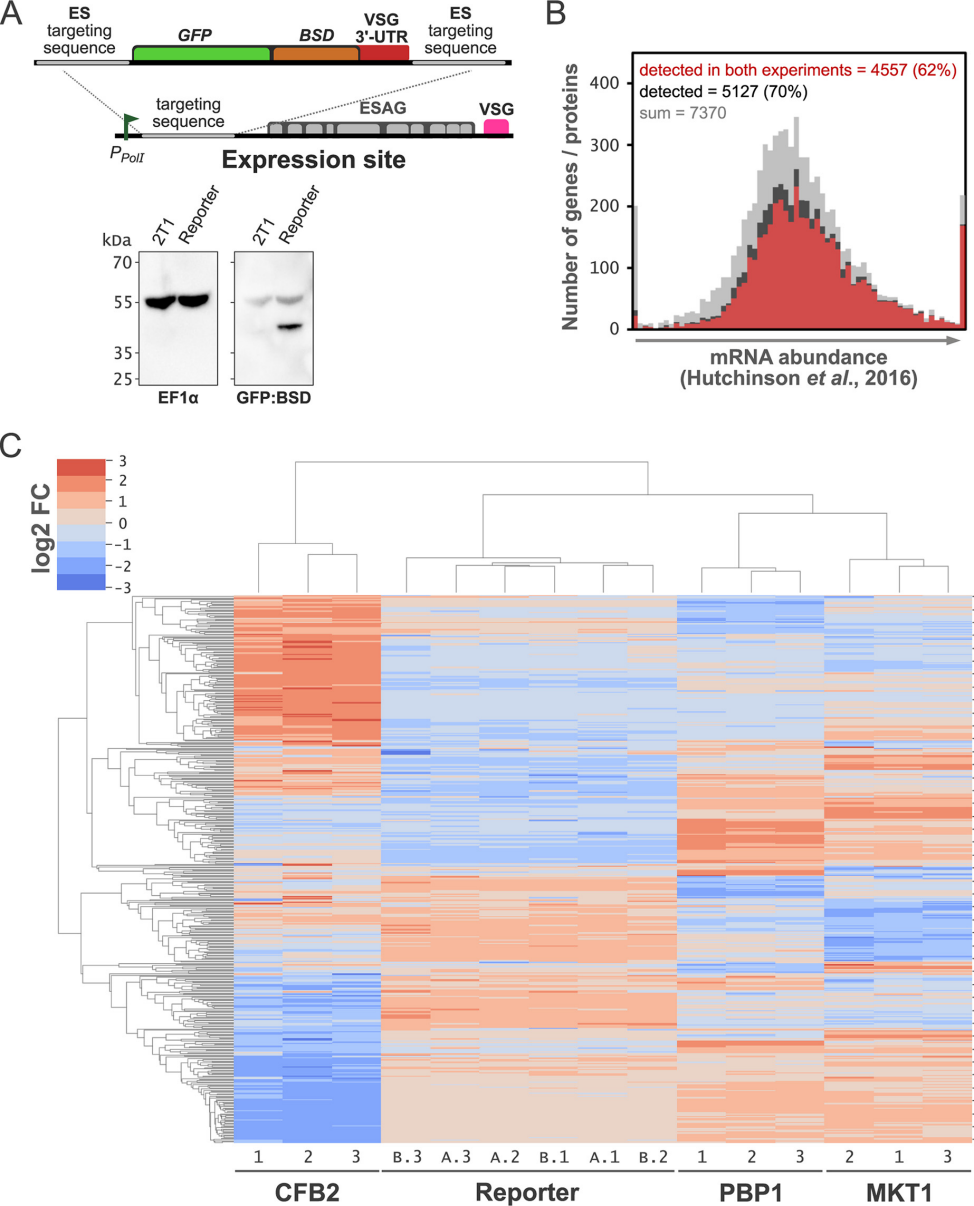
Proteomic profiles and clustering of mRBP knockdowns. (A, top) Schematic representation of the cassette harboring the reporter gene targeted to the VSG expression site (ES) promoter region. The cassette contains a hybrid gene that includes *GFP* and the blasticidin resistance gene (*BSD*) together with the VSG 3′ UTR. ESAG, expression site-associated genes. (Bottom) Western blotting with anti-EF1α antibody and rehybridization of the same membrane with mouse anti-GFP of proteins obtained from cell extracts containing ∼1 × 10^7^ cells of the parental 2T1 strain or the reporter strain. (B) Plot showing those proteins detected by proteomic analysis relative to those mRNAs detected by transcriptome analysis (19). (C) Clustering of differentially expressed proteins. Samples were run in two batches and on separate occasions: the first one including the MKT1 and PBP1 knockdown strains and the second one including the CFB2 knockdown strain. In both batches, the reporter strain was included as a further reference control. After directDIA analysis, a total of 466 protein groups were differentially expressed in the TET-induced samples with respect to the uninduced samples under a threshold false discovery rate (FDR) of <0.01 and a fold change (FC) of less than −2 (underrepresented) or greater than 2 (overrepresented) in at least one knockdown strain (see Data Set S1 in the supplemental material). These 466 differentially expressed proteins were clustered according to the protein abundance values for the three RNAi mutant replicates (MKT1.1 to .3, PBP1.1 to .3, and CFB2.1 to .3) and the reporter strain from the first (A.1 to .3) and second (B.1 to .3) batches.

We selected proteomic profiling to assess the roles of the candidate VSG regulatory RNA binding proteins CFB2, MKT1, and PBP1 in more detail. This approach can reveal altered profiles following knockdown that may not be detected using transcriptomic approaches, for example. Since we were particularly interested in VSG expression control, we first added a reporter gene to the experimental strain. Disruption of VSG expression, by knockdown of the VSG transcript itself, was previously reported to result in rapid cell cycle arrest without a detectable reduction in VSG abundance, as assessed using anti-VSG antibodies (8). This may be explained by a remarkably low rate of VSG turnover (16, 17). To assess VSG controls that may, due to slow turnover, fail to register a change in VSG protein abundance, we assembled a strain expressing a reporter under the control of a (181-bp) *VSG* 3′ untranslated region (UTR) (Fig. 2A). This sequence contains a conserved 16-mer motif (TGATATATTTTAACAC) that is thought to bind a specific positive regulator (6). The *VSG* 3′-UTR-associated reporter was targeted to the region immediately downstream of the *VSG* expression site promoter and comprised green fluorescent protein (GFP) fused to an antibiotic-selectable marker, the blasticidin resistance gene (BSD) (Fig. 2A). The expression of the reporter was confirmed by protein blotting (Fig. 2A). Thus, the reporter strain provides a readout of *VSG* 3′-UTR-specific controls. In addition, even moderate impacts on the VSG itself may be detected using quantitative proteomics, which is typically more sensitive than quantitative protein blotting.

10.1128/msphere.00069-22.2 DATA SET S1 Mass spectrometry data and oligonucleotides used in this study. Download Data Set S1, XLSX file, 2.5 MB. Copyright © 2022 Bravo Ruiz et al. 2022 Bravo Ruiz et al. https://creativecommons.org/licenses/by/4.0/ This content is distributed under the terms of the Creative Commons Attribution 4.0 International license.

We induced CFB2, MKT1, or PBP1 knockdown in the GFP:BSD reporter strain and prepared protein lysates for proteomic analysis, with triplicate samples from the parental strain, uninduced cells, and cells induced for 24 h. Specifically, we used directDIA (Biognosys AG) (an implementation of a library-free data-independent acquisition [DIA] method) mass spectrometry (MS), which provides accurate label-free quantification (LFQ) and deep proteome coverage (18). The data were assessed using a predicted proteome for the well-annotated T. brucei 927 genome reference strain and the VSG expression sites from the T. brucei Lister 427 strain. Samples were separated into two batches, containing the MKT1 and PBP1 knockdowns in the first batch and the CFB2 knockdown in the second. In both cases, the reporter strain was added as a reference. To assess the depth of proteome coverage, we compared the proteomic data sets from the parental strain with our previously reported transcriptomic data set (19), including data for 7,370 nonredundant genes, the vast majority of annotated genes in the T. brucei genome. Seventy percent of the cognate proteins (5,127 proteins) were detected in one or the other from two independent proteomics experiments using the reference strain, while 62% were detected in both experiments (Fig. 2B), representing an excellent depth of quantitative proteome coverage. As expected, we detected a higher proportion of those proteins derived from the more abundant transcripts.

Next, we used cluster analysis to compare the full data set derived from all 24 mass spectrometry runs. Cluster analysis of differentially expressed proteins revealed excellent correspondence and clustering of triplicate runs from the reference strain and the knockdowns (Fig. 2C). Importantly, sets of triplicate runs for the parental strain also displayed excellent correspondence and clustering (Fig. 2C; see also Fig. S1 in the supplemental material). In contrast, each knockdown yielded a distinct pattern of differential expression, although the expression patterns associated with MKT1 or PBP1 knockdown appeared to display some similarities (Fig. 2C). Thus, the proteomics approach used here provides deep quantitative coverage that is both consistent and reproducible. These data can be searched and browsed using an interactive, open-access, online application available at https://gustavo-e64.pages.dev (MKT1), https://gustavo-e84.pages.dev (PBP1), and https://gustavo-e146.pages.dev (CFB2).

10.1128/msphere.00069-22.1 FIG S1 Assessment of reproducibility in the proteomics analysis. A pairwise comparison of the protein intensity values (log_2_) of the GFP reporter strain replicates in the first (GFPA.1 to .3) and second (GFPB.1 to .3) batches was performed. The top diagonal visualizes the protein intensity value (log_2_) distribution with histogram counts. Download FIG S1, PDF file, 0.3 MB. Copyright © 2022 Bravo Ruiz et al. 2022 Bravo Ruiz et al. https://creativecommons.org/licenses/by/4.0/ This content is distributed under the terms of the Creative Commons Attribution 4.0 International license.

### CFB2 positively regulates VSG expression.

**FIG 3:**
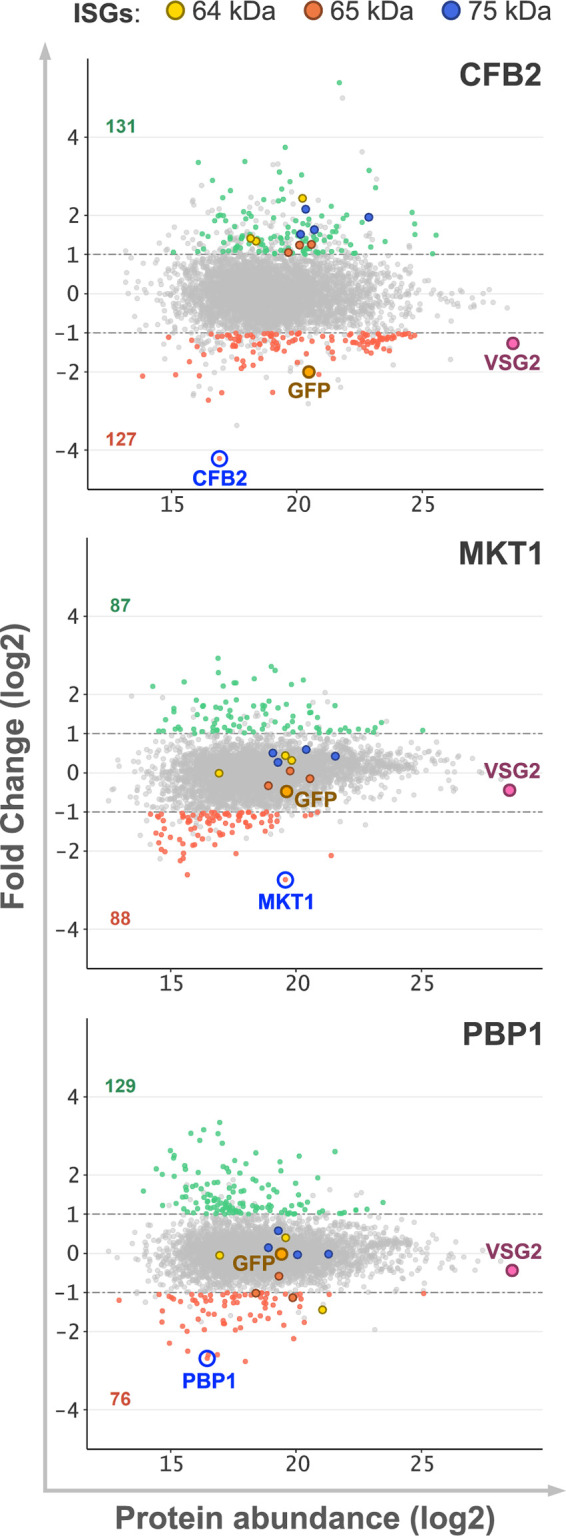
Assessment of candidate VSG regulator knockdowns using quantitative proteomics. The MA (log ratio versus mean average) plots show protein abundance changes following CFB2, MKT1, or PBP1 knockdown after 24 h relative to uninduced samples for all proteins quantified by directDIA analysis (see Data Set S1 in the supplemental material). Green and red dots represent overrepresented (FC of greater 2; false discovery rate [FDR] of <0.01) and underrepresented (FC of less than −2; FDR of <0.01) proteins in each case. Gray dots represent all other proteins detected. Green and red numbers indicate the total proteins over- or underrepresented in each knockdown strain. The target knockdown protein (blue label and circle), the active VSG (VSG2) (purple label and dot), the GFP:BSD reporter including the VSG 3′ UTR (GFP) (orange label and dot), and the invariant surface glycoproteins (ISGs) are indicated.

Having established that our proteomic analysis yielded deep and reliable quantitative expression profiles following RBP knockdown, we next assessed each profile in more detail. Following CFB2, MKT1, or PBP1 knockdown, approximately 200 proteins were differentially expressed in each case (>2-fold; false discovery rate [FDR] of <0.01), as indicated by plotting the protein abundance against the fold change (FC) (Fig. 3). Each profile confirmed the efficient knockdown of the targeted RBP. Indeed, CFB2, MKT1, and PBP1 were >5-fold depleted in each case and ranked first (CFB2 and MKT1) or second (PBP1) in terms of the most depleted protein in their respective knockdowns (Fig. 3). CFB2 depletion was the most pronounced, possibly explaining the more pronounced loss of fitness described above (Fig. 1B). We next looked at VSG-2 expression and the expression of the GFP:BSD reporter under the control of a *VSG* 3′ UTR and observed a striking depletion of both proteins specifically associated with CFB2 knockdown (Fig. 3). These results are consistent with the view that CFB2 directly regulates VSG expression in a *VSG* 3′-UTR-dependent manner (6).

Our results indicate that VSG-2, which typically forms a dense surface coat, is depleted >2-fold following CFB2 knockdown, suggesting that the VSG surface coat is substantially compromised (Fig. 3). This is in contrast to the minimally perturbed VSG coat reported previously following direct *VSG* mRNA knockdown (8). Since the VSG coat is perturbed following CFB2 knockdown, we asked whether the reduction in molecular crowding on the cell surface plasma membrane allowed the increased abundance of invariant surface glycoproteins (ISGs). Consistent with this view, the expression levels of ISG64, ISG65, and ISG75 were all specifically increased following CFB2 knockdown (Fig. 3). Notably, these ISGs were similarly upregulated following the depletion of a component of the exocyst, possibly also reflecting VSG coat perturbation, due to exocytosis and recycling defects in the latter case (20).

### PBP1 stabilizes a multisubunit CFB2-associated complex.

**FIG 4:**
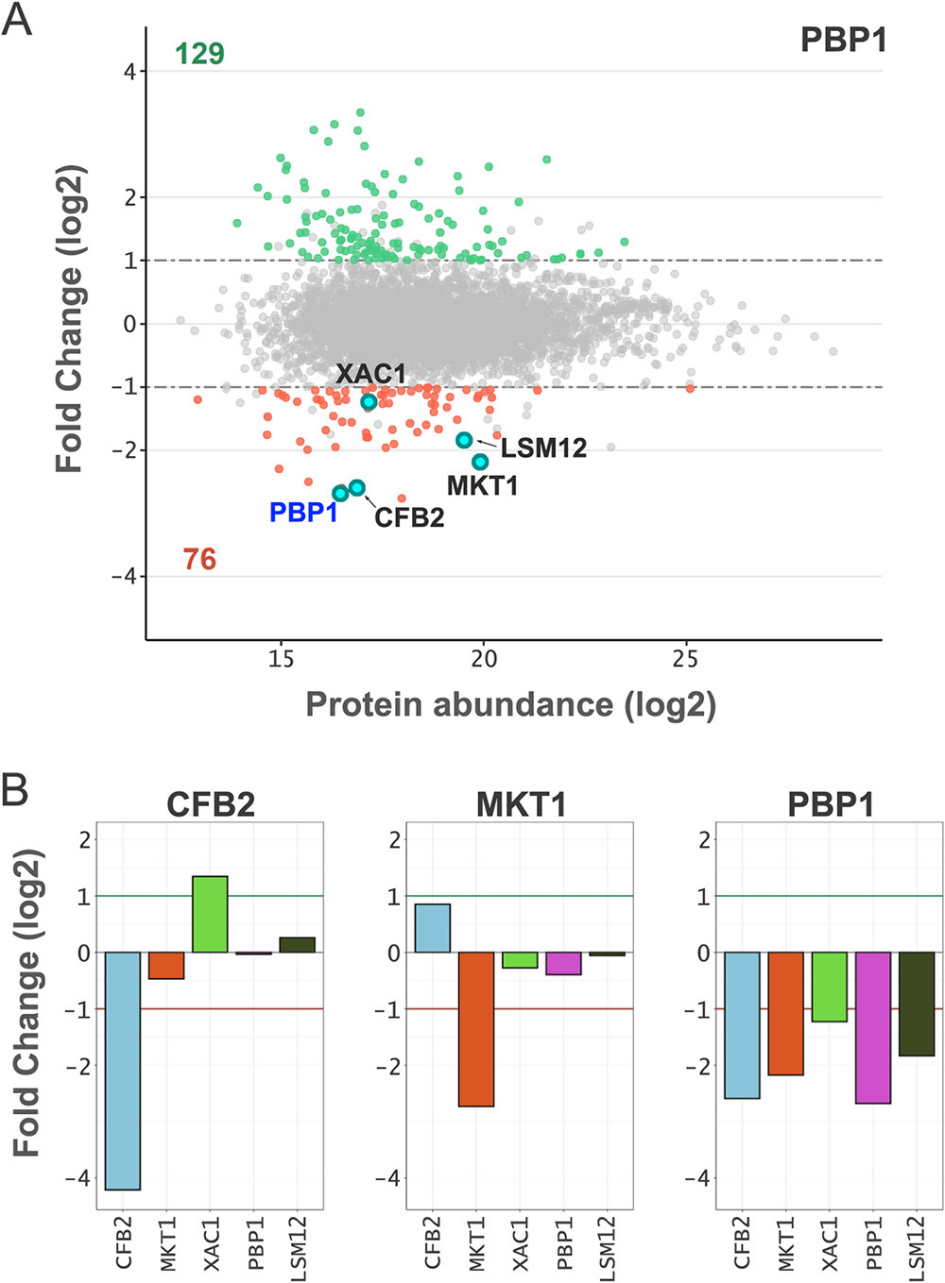
Quantitative proteomic assessment of the PBP1 complex. (A) MA plot showing protein abundance changes following PBP1 knockdown after 24 h relative to uninduced samples for all proteins quantified by directDIA analysis (see Data Set S1 in the supplemental material). Proteins thought to be associated with PBP1 are represented as cyan dots, and PBP1 is labeled in blue. Other details are the same as those described in the Fig. 3 legend. (B) Bar plots showing fold changes for proteins thought to be associated with PBP1 in each knockdown strain. The fold change thresholds are indicated by green (greater than 2) and red (less than −2) lines.

CFB2 is thought to interact with a number of other proteins as part of an mRBP complex, including MKT1 and PBP1 and also LSM12 and XAC1 (6). Remarkably, all four additional components of this complex are significantly depleted following PBP1 knockdown (Fig. 4A and B), but none of the other components are significantly depleted following either CFB2 or MKT1 knockdown (Fig. 4B). We conclude that although these proteins interact, only PBP1 specifically impacts the abundance of other components of the complex. The mechanism is most likely through protein binding increasing the stability of the individual components. Nevertheless, we cannot rule out control via PBP1 binding the cognate mRNAs.

The pentameric mRBP complex described above is thought to interact with EIF4E6/EIF4G5 and the poly(A) binding protein PABP2 in association with VSG mRNA (6). None of the latter three proteins is significantly depleted following PBP1 knockdown. PABP1 and PABP2 are both notably depleted following CFB2 knockdown (>1.6-fold), however (see below).

### CFB2 knockdown impacts ribosomal protein expression.

**FIG 5:**
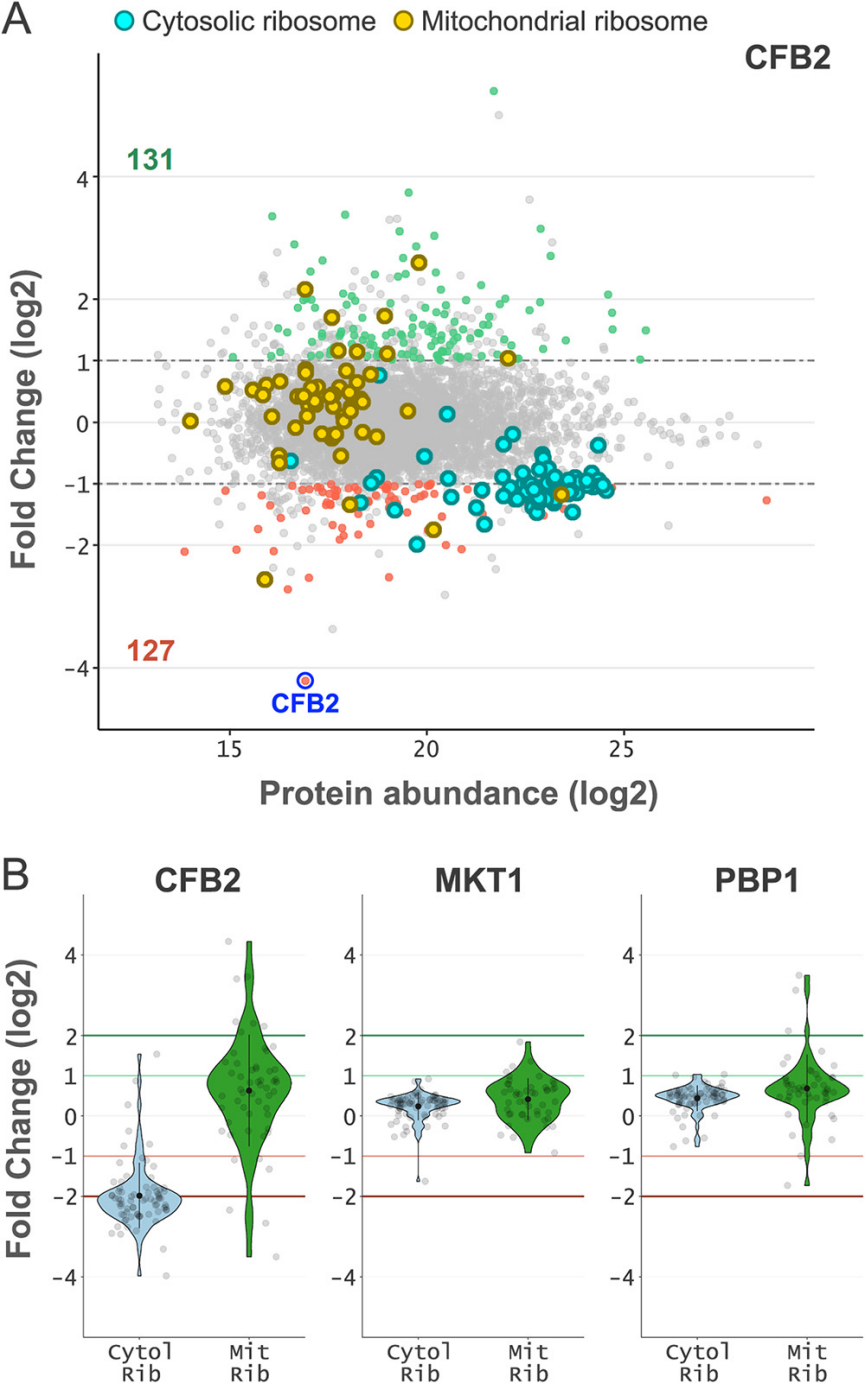
CFB2 knockdown impacts ribosomal protein expression. (A) MA plot showing the protein abundance changes following CFB2 knockdown after 24 h relative to uninduced samples for all proteins quantified by directDIA analysis (see Data Set S1 in the supplemental material). Other details are the same as those described in the Fig. 3 legend. Components of the cytosolic and mitochondrial ribosomes are represented as cyan and yellow dots, respectively. (B) Violin plots showing fold changes for cytosolic and mitochondrial ribosome components in each knockdown strain. The internal black dot represents the mean fold change, and the black line indicates the standard deviation (SD). The fold change thresholds are indicated by green (greater than 2) and red (less than −2) lines.

Blocking VSG synthesis triggers a general arrest in translation initiation (21), but the mechanism remains unknown. We observed a striking and specific depletion of ribosomal proteins following CFB2 knockdown (Fig. 5A). This impact was specific to components of the cytoplasmic ribosome, as opposed to the mitochondrial ribosome, and was not observed following either MKT1 or PBP1 knockdown (Fig. 5B). Notably, cytoplasmic ribosomal proteins are among the most abundant proteins in the cell, such that an average >2-fold depletion of >80 of these proteins, as observed here, represents a major remodeling of the proteome. CFB2 may control the expression of the cytoplasmic ribosomal subunits by interacting with the cognate transcripts or by interacting with the ribosomes themselves. Notably, in this regard, the abundances of transcripts encoding cytoplasmic and ribosomal proteins were 91% ± 4% and 94% ± 8%, respectively, following 9 h of CFB2 knockdown (6). Alternatively, the depletion of the poly(A) binding proteins (see above) may negatively impact global translation. Whichever mechanism operates, cytoplasmic ribosomal protein depletion following CFB2 knockdown reveals a connection between VSG expression and the core translation machinery. We suggest that this connection also underpins translation arrest when VSG expression is perturbed directly.

### Proteomic profiling reveals links between mRBPs and mitosis and cytokinesis defects.

**FIG 6:**
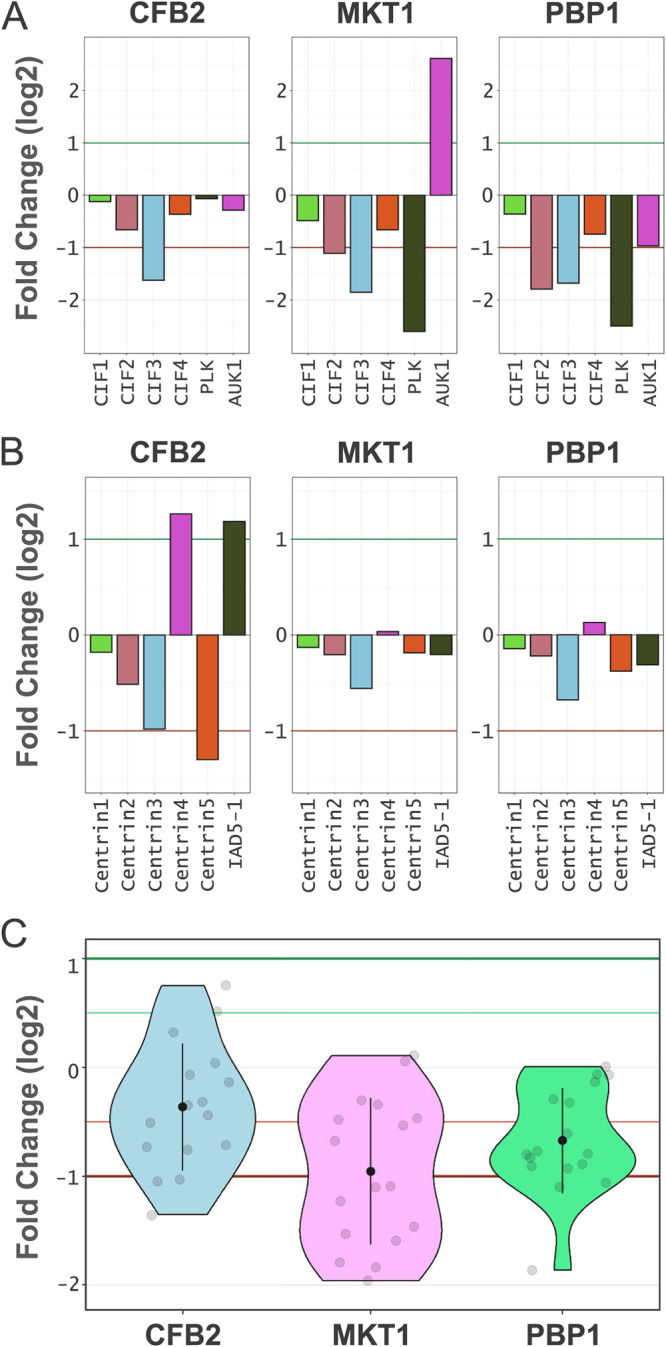
Links to cell cycle defects. The plots show protein fold changes following CFB2, MKT1, or PBP1 knockdown after 24 h relative to uninduced samples for selected proteins quantified by directDIA analysis (see Data Set S1 in the supplemental material). The fold change thresholds are indicated by green (greater than 2) and red (less than −2) lines. (A) Bar plots showing proteins known to be involved in cytokinesis. (B) Bar plots showing T. brucei centrin proteins and centrin3-associated inner-arm dynein 5-1 (IAD5-1). (C) Violin plot showing the kinetoplastid kinetochore proteins. The internal black dot represents the mean fold change, and the black line indicates the SD.

All three candidate VSG regulators analyzed here were selected on the basis that knockdown was associated with overrepresentation at the G_2_M phase of the cell cycle in a genome-scale screen (Fig. 1A), a phenotype confirmed here for all three knockdowns (Fig. 1C). As with the connection between the ribosome and translation arrest detailed above, we identified a signature in the proteomic profiles that revealed potential connections to the common cytokinesis defect and also to the pronounced endoreduplication with continued mitosis that was specific to the CFB2 knockdown. For this analysis, we focus on cohorts of differentially expressed proteins following knockdown that were previously linked to cytokinesis or mitosis defects (Fig. 6).

First, we found that cytokinesis initiation factor 3 (CIF3) (22) was underrepresented in all three knockdowns, potentially explaining the cytokinesis defect observed, while CIF2 and polo-like kinase (PLK) were also underrepresented following MKT1 or PBP1 knockdown (Fig. 6A). In contrast, the aurora kinase AUK1 (23, 24) is specifically overrepresented following MKT1 knockdown. We also found that centrin expression was specifically disrupted following CFB2 knockdown (Fig. 6B), potentially explaining the continued endoreduplication and mitosis. In T. brucei, centrin3 forms a complex with the inner-arm dynein heavy chain IAD5-1, and disruption of this complex yields endoreduplicated and multinucleated cells and also reduced CIF3 expression (25, 26), which is similar to what we observe here following CFB2 knockdown (Fig. 1C and D). Two additional centrins, centrin4 (27) and centrin5 (28), are also specifically perturbed following CFB2 knockdown (Fig. 6A).

Endoreduplication and continued rounds of mitosis specifically following CFB2 knockdown may also be linked to differential kinetoplastid kinetochore protein (KKT) expression (Fig. 6C). These cell cycle-regulated proteins (29) are required for chromosome segregation during mitosis (30, 31) and are retained at a higher level following CFB2 knockdown than following MKT1 or PBP1 knockdown. Thus, higher KKT expression levels may facilitate continued rounds of mitosis observed primarily following CFB2 knockdown (Fig. 1D).

### Concluding remarks.

We used previous genome-scale knockdown screening data reporting loss of fitness (10) and precytokinesis arrest (11) to prioritize three candidate mRBP VSG regulators, namely, CFB2, MKT1, and PBP1. Quantitative proteomic analysis following the depletion of each mRBP revealed the significantly reduced expression of VSG and a reporter under the control of the *VSG* 3′ UTR, specifically following CFB2 knockdown. All three proteins interact in association with the VSG transcript, however (6), and we find that the mRBP complex is specifically destabilized following PBP1 knockdown. We observed approximately 100 further proteins either overrepresented or underrepresented following the knockdown of either CFB2, MKT1, or PBP1, and the individual profiles reveal specific connections to translation arrest (21) and cytokinesis defects (8), also observed following VSG knockdown. These findings reveal further insight into how CFB2 impacts VSG expression. The results also reveal the codestabilization of components of the multisubunit CFB2-associated complex following PBP1 knockdown and a role for CFB2 in controlling ribosome abundance. Finally, we discuss connections uncovered between these mRBPs and the machinery driving mitosis and cytokinesis.

## MATERIALS AND METHODS

### T. brucei growth and manipulation.

Bloodstream-form T. brucei Lister 427 cells were routinely grown in Hirumi's modified Iscove's medium (HMI-11) medium at 37°C with 5% CO_2_ (32) and the appropriate antibiotics. Cumulative growth curves were generated from cultures seeded at 10^5^ cells/mL in HMI-11 medium or under inducing conditions for RNAi knockdown, with 1 μg/mL tetracycline. Cells were counted on a hemocytometer every 24 h and diluted as necessary.

### Construct and strain assembly.

To generate a VSG 3′-UTR reporter strain, a construct was obtained by fusion PCR (33), yielding a hybrid gene consisting of green fluorescent protein (GFP) and the blasticidin resistance gene (BSD), including the VSG 3′ UTR (181 bp) and flanked by ∼600-bp regions homologous to the T. brucei Lister 427 expression site promoter region (Fig. 2A). For this, two initial PCR amplicons were obtained: the first amplicon contained the 5′ expression site-flanking region and the GFP:BSD hybrid gene (without any 3′ UTR) obtained using a pESp_GFP:BSD cassette as the template and the M13F and p18 oligonucleotides (see Data Set S1 in the supplemental material), and the second amplicon contained the VSG 3′ UTR and the 3′ expression site-flanking region obtained using a synthetic NPT_VSG-3UTR_ESP plasmid (Invitrogen) as the template and the p19 and M13R oligonucleotides (Data Set S1). Oligonucleotides p18 and p19 include 30-bp-overhang 5′ tails homologous to each other to merge both amplicons by fusion PCR. For fusion PCR, ∼100 ng/μL of each fragment was combined with the other components in a standard PCR mix but without primers (tails from oligonucleotides p18 and p19 served as primers). Following 8 fusion PCR cycles (denaturalization, annealing, and extension), a final standard PCR was performed using nested oligonucleotides p16 and p17 (Data Set S1) and 2 to 5 μL of the fusion PCR product. Following agarose gel electrophoresis, the product was purified using a QIAquick gel extraction kit (Qiagen).

For gene-specific knockdown RNAi constructs, target gene fragments of 400 to 600 bp were amplified and cloned into the pRPa^iSL^ plasmid for the generation of stem-loop double-stranded RNA (dsRNA) as the trigger for RNAi (34). The necessary oligonucleotides (Data Set S1) were designed using the RNAit tool (35) (https://dag.compbio.dundee.ac.uk/RNAit/). Before transfection, knockdown constructs were linearized using AscI (New England BioLabs).

The reporter strain was obtained after transfection of the T. brucei Lister 427 2T1 strain with the construct containing the GFP:BSD reporter gene and the VSG 3′ UTR. The 2T1 strain contains a tagged rRNA locus for the site-specific integration of the different RNAi constructs (34). The reporter strain was accordingly used in three independent new transfections to introduce the linearized RNAi constructs at the tagged rRNA locus. For each transfection, 2.5 × 10^7^ cells, resuspended in homemade transfection buffer (36) with approximately 10 μg of construct DNA were electroporated as described previously (37), using a Nucleofector system (Lonza) set on program Z-001. After 4 to 6 h, transformants were cloned by limiting dilution and selected with blasticidin (reporter strain) (10 μg/mL) or hygromycin (RNAi constructs) (2.5 μg/mL).

### Protein blotting.

Cell extracts from ∼5 × 10^7^ cells were harvested for protein extraction. Protein samples were run according to standard protein separation procedures and protocols, using 4 to 12% precast SDS-PAGE gels (NuPAGE; Invitrogen). For GFP:BSD detection, a mouse anti-GFP (1:1,000) (Roche) primary antibody was used. Mouse anti-EF1α (1:20,000) (Millipore) primary antibody was used for a loading control. As a secondary antibody, goat anti-mouse coupled to horseradish peroxidase (1:2,000) (Bio-Rad) was used. Blots were developed using an enhanced chemiluminescence kit (Amersham) according to the manufacturer’s instructions. Densitometry was performed using a ChemiDoc XRS^+^ system (Bio-Rad).

### Flow cytometry and microscopy.

Approximately 1 × 10^7^ TET-induced (24 h) and uninduced cells from each RNAi strain were harvested and fixed by adding 1% formaldehyde in supplemented phosphate-buffered saline (PBS) (1× PBS, 5 mM EDTA, 1% fetal bovine serum [FBS]) dropwise and with regular shaking. The cells were incubated for 10 min at room temperature, washed in 1 mL of supplemented PBS, and then resuspended in 250 μL of supplemented PBS. Samples were stored at 4°C in the dark until further processing. Before flow cytometry analysis, cells were centrifuged for 10 min at 1,000 × *g* and resuspended in 1 mL of supplemented PBS containing 0.01% Triton X-100 (Sigma-Aldrich). The cells were incubated for 30 min at room temperature, centrifuged for 10 min at 700 × *g*, and washed once in 1 mL of supplemented PBS. Cells were then resuspended in 400 μL supplemented PBS with 10 μg/mL propidium iodide (Sigma-Aldrich) and 100 μg/mL RNase A (Sigma-Aldrich) and then incubated at 37°C in the dark for 45 min. Samples were then run on a BD FACSCanto flow cytometer for cell cycle analysis. FlowJo v10 was used for data analysis and visualization.

For microscopy, approximately 1 × 10^6^ TET-induced and uninduced cells were fixed in 1% formaldehyde in 1 mL of culture medium at 37°C for 5 min and then at room temperature for 10 min. Cells were rinsed twice in PBS for 10 min, with spins at 1,000 × *g*, and then resuspended in 600 μL of ice-cold 1% bovine serum albumin (BSA) in water. Cells were dried on microscopy slides overnight at room temperature, rehydrated in PBS for 5 min, and then mounted in Vectashield antifade mounting medium with 4′,6-diamidino-2-phenylindole (DAPI) (Vector) under a coverslip. Cells were visualized at a ×63 magnification with oil immersion under a Zeiss Axiovert 200M microscope with Zen pro software (Zeiss) in the DAPI and phase channels. The cell cycle stages of at least 100 cells were identified and counted manually.

### Mass spectrometry.

Proteins for each RNAi strain were extracted from ∼5 × 10^7^ cells after growth for 24 h under standard conditions with or without TET. Cell extracts were resuspended in 100 μL of a solution containing 5% SDS and 100 mM triethylammonium bicarbonate and submitted to the Fingerprints Proteomics Facility at the University of Dundee to be analyzed by directDIA using Spectronaut software (Biognosys). Triplicate samples of each RNAi strain were submitted for proteomics analysis in two batches: the first including the MKT1 and PBP1 RNAi strains and the second including the CFB2 RNAi strain. The reporter strain was included in triplicate in both batches as a further control. Samples were processed using trypsin (μBCA [bicinchoninic acid], strap processed, quality controlled, and peptide quantified) using 200 μg from each sample, and the final peptide quantification yielded between 72 and 120 μg. For liquid chromatography-mass spectrometry (LC-MS) analysis, 1.5 μg of each sample was injected onto a nanoscale C_18_ reverse-phase chromatography system (UltiMate 3000 RSLC [rapid-separation liquid chromatography] nano; Thermo Scientific) and then electrosprayed into a Q Exactive HF-X mass spectrometer (Thermo Scientific). For liquid chromatography, buffers were as follows: buffer A was 0.1% (vol/vol) formic acid in MilliQ water, and buffer B was 80% (vol/vol) acetonitrile and 0.1% (vol/vol) formic acid in MilliQ water. Samples were loaded at 10 μL/min onto a trap column (100-μm by 2-cm PepMap nanoViper C_18_ column, 5 μm, 100 Å; Thermo Scientific) equilibrated in 0.1% trifluoroacetic acid (TFA). The trap column was washed for 3 min at the same flow rate with 0.1% TFA and then switched inline with a Thermo Scientific resolving C_18_ column (75-μm by 50-cm PepMap RSLC C_18_ column, 2 μm, 100 Å). The peptides were eluted from the column at a constant flow rate of 300 nL/min with a linear gradient from 3% buffer B to 6% buffer B in 5 min and then from 6% buffer B to 35% buffer B in 115 min and, finally, to 80% buffer B within 7 min. The column was then washed with 80% buffer B for 4 min and reequilibrated in 35% buffer B for 5 min. Two blanks were run between each sample to reduce carryover. The column was kept at a constant temperature of 40°C.

The data were acquired using an easy-spray source operated in positive mode with spray voltage at 2,500 kV and the ion transfer tube temperature at 250°C. The MS system was operated in DIA mode. A scan cycle comprised a full MS scan (*m/z* range from 350 to 1,650), with the RF lens at 40%, the automatic gain control (AGC) target set to custom, the normalized AGC target set at 300, the maximum injection time mode set to custom, the maximum injection time at 20 ms, and source fragmentation disabled. The MS survey scan was followed by tandem MS (MS/MS) DIA scan events using the following parameters: multiplex ions set to false; collision energy mode set to stepped; collision energy type set to normalized; high-energy collisional dissociation (HCD) collision energies set to 25.5, 27, and 30; orbitrap resolution at 30,000; first mass at 200; radio frequency lens at 40; AGC target set to custom; normalized AGC target at 3,000; and maximum injection time of 55 ms. Data for both MS and MS/MS scans were acquired in profile mode. Mass accuracy was checked before the start of sample analysis.

### Proteome analysis. (i) directDIA analysis.

Spectronaut directDIA analysis was carried out using version 15.4.210913.50606 (Biognosys). Trypsin was set as the enzyme with a maximum of two missed cleavages. Fixed modification was set for carbamidomethyl, and variable modifications were set for protein N-terminal acetylation, oxidation of methionine, dioxidation of methionine and tryptophan, glutamine to pyroglutamate, and deamidation of asparagine and glutamine. The identifications were filtered at an FDR of 1% at both the peptide and protein levels. The protein LFQ method was set to Quant 2.0, and data filtering was set to *Q* value. All other settings were set to default.

Although we used the T. brucei Lister 427 strain, for protein identifications, we used the protein database of the T. brucei 927 reference strain, obtained from TriTrypDB (https://tritrypdb.org/tritrypdb/app/), combined with predicted proteins from the VSG expression sites from the Lister 427 strain (38).

### (ii) Differential protein abundance.

Data analysis was performed using custom Python and R scripts, using the SciPy ecosystem of open-source software libraries (39). The exact software versions of the environment used for the analysis are listed in the pkg_version.txt file at https://github.com/mtinti/Gustavo_DIA_RBP. A protein group pivot table was exported from the output of the CellRanger analysis. The protein groups identified as single hits were considered missing values. Protein groups with more than four missing values were excluded from the analysis. The missing values were imputed using missForest (40) after log_2_ transformation of the data. The differential expression analysis was performed with the limma package (41) using the tetracycline-induced samples versus the uninduced samples. FDR values were computed with the toptable function in limma.

### (iii) Clustering analysis.

We extracted the protein abundance values from the first batch, MKT1, PBP1, and the control reporter strain (“GFPA”), and the second batch, CFB2 and the control reporter strain (“GFPB”), using the CellRanger pivot tables. We then used the removeBatchEffect function in limma (41) or the ComBat function in the R sva package (42). We used the coefficients of variation between the control strain experiments (GFPA.1 to .3 and GFPB.1 to .3) to evaluate the results. The removeBatchEffect function was chosen over ComBat for providing moderately lower coefficients of variation. After removing the batch effect, we selected differentially abundant proteins from the MKT1, PBP1, and CFB2 data sets. To this aim, we used a threshold of <0.01 for the FDR and a log_2_ fold change of less than −1, or greater than 1, using the analysis described in the paragraph above. We further removed from the analysis protein groups with any number of missing values. This allowed the selection of 466 protein groups in common among the MKT1, PBP1, and CFB2 data sets that showed differential abundance values in at least one experiment. The log_2_ abundance values were z-score transformed raw-wise and used for clustering analysis using the clustermap function in the seaborn Python package (https://seaborn.pydata.org/).

### Data availability.

The mass spectrometry proteomics data have been deposited to the ProteomeXchange Consortium via the PRIDE (43) partner repository with data set accession number PXD031351 (https://www.ebi.ac.uk/pride/archive/projects/PXD031351). The code to reproduce the analysis pipeline was deposited in GitHub (https://github.com/mtinti/Gustavo_DIA_RBP) and archived in zenodo (https://doi.org/10.5281/zenodo.5761826).
