# Supplementary figures and images for: Control of Variant Surface Glycoprotein Expression by CFB2 in Trypanosoma brucei and Quantitative Proteomic Connections to Translation and Cytokinesis
Source: mSphere. 2022 Mar 21;7(2):e00069-22. doi: 10.1128/msphere.00069-22 (PMC9044945; doi:10.1128/msphere.00069-22)

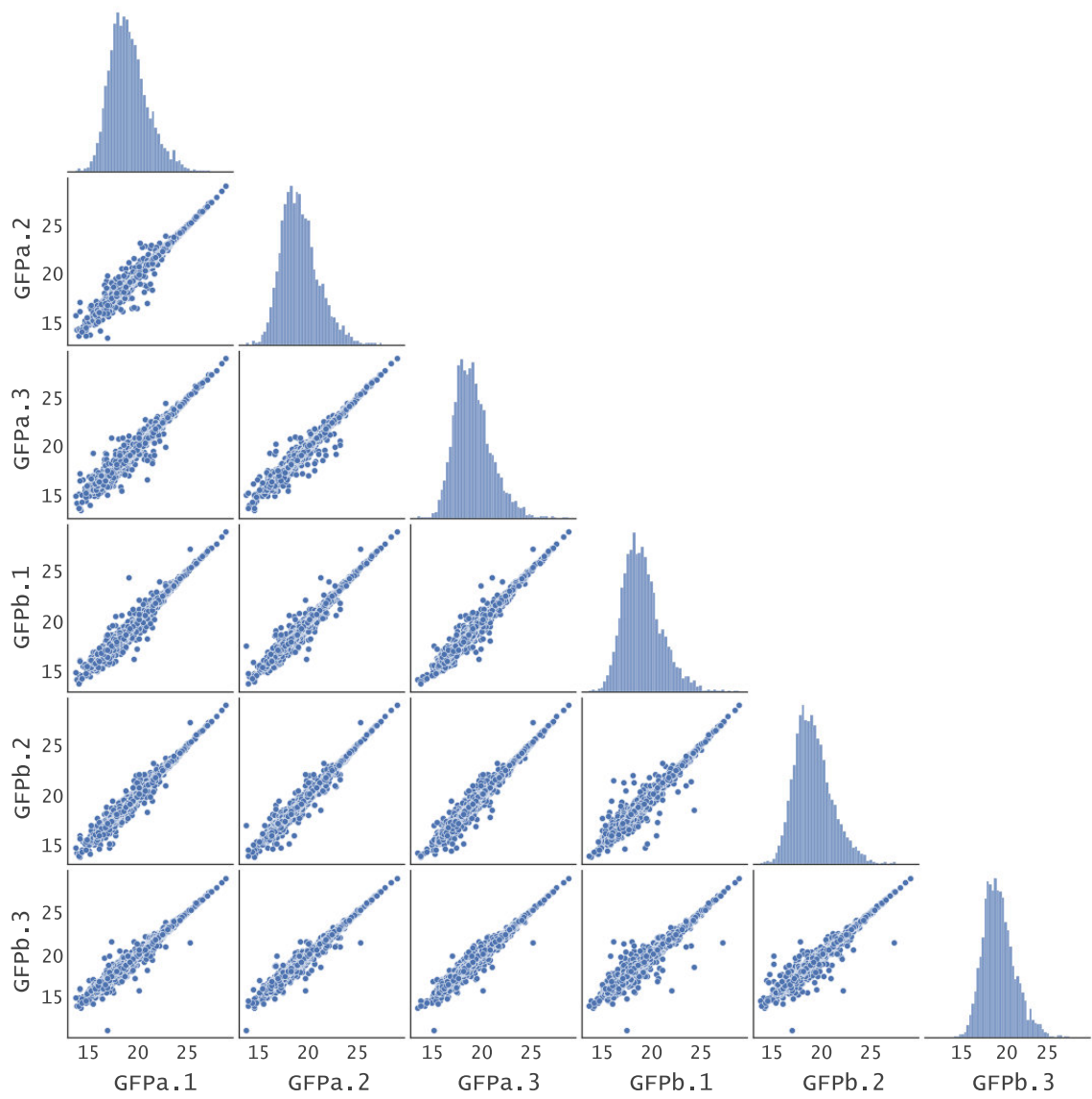

Supplement: FIG S1 [file msphere.00069-22-sf001.pdf]
